# Purification, Physicochemical Properties, and Antioxidant Activities of Two Low-Molecular-Weight Polysaccharides from *Ganoderma leucocontextum* Fruiting Bodies

**DOI:** 10.3390/antiox10071145

**Published:** 2021-07-20

**Authors:** Xiong Gao, Jiayi Qi, Chi-Tang Ho, Bin Li, Yizhen Xie, Shaodan Chen, Huiping Hu, Zhongzheng Chen, Qingping Wu

**Affiliations:** 1 State Key Laboratory of Applied Microbiology Southern China, Guangdong Provincial Key Laboratory of Microbial Safety and Health, Institute of Microbiology, Guangdong Academy of Sciences, Guangzhou 510070, China; gaoxiong@gdim.cn (X.G.); xieyz@gdim.cn (Y.X.); chensd@gdim.cn (S.C.); huhp@gdim.cn (H.H.); 2 Guangdong Yuewei Edible Fungi Technology Co. Ltd., Guangzhou 510663, China; 3 Department of Bioengineering, College of Food Science, South China Agricultural University, 483 Wushan Street, Tianhe District, Guangzhou 510642, China; qijiayi0511@stu.scau.edu.cn (J.Q.); bli@scau.edu.cn (B.L.); 4 Department of Food Science, Rutgers University, 65 Dudley Road, New Brunswick, NJ 08901, USA; ctho@sebs.rutgers.edu; 5 Guangdong Provincial Key Laboratory of Nutraceuticals and Functional Foods, Department of Food Science, College of Food Science, South China Agricultural University, Guangzhou 510642, China

**Keywords:** *Ganoderma leucocontextum*, polysaccharides, physicochemical property, antioxidant activity

## Abstract

Two low-molecular-weight polysaccharides (GLP-1 and GLP-2) were purified from *Ganoderma leucocontextum* fruiting bodies, and their physicochemical properties and antioxidant activities were investigated and compared in this study. The results showed that GLP-1 and GLP-2 were mainly composed of mannose, glucose, galactose, xylose, and arabinose, with weight-average molecular weights of 6.31 and 14.07 kDa, respectively. Additionally, GLP-1 and GLP-2 had a similar chain conformation, crystal structure, and molecular surface morphology. Moreover, GLP-1 exhibited stronger antioxidant activities than GLP-2 in five different assays: 2,2′-azino-bis(3-ethylbenzthiazoline-6-sulfonic acid) (ABTS), hydroxyl radical, superoxide anion radical, ferric reducing antioxidant power (FRAP), and oxygen radical antioxidant capacity (ORAC). The main linkage types of GLP-1 were found to be →4)-*α*-D-Glc*p*-(1→, →4)-*β*-D-Glc*p*-(1→, →3)-*β*-D-Glc*p*-(1→, →6)-*β*-D-Gal*p*-(1→, →6)-*α*-D-Glc*p*-(1→, →4,6)-*α*-D-Glc*p*-(1→, and Glc*p*-(1→ by methylation analysis and nuclear magnetic resonance (NMR) spectroscopy. In addition, GLP-1 could protect NIH3T3 cells against tert-butyl hydroperoxide (tBHP)-induced oxidative damage by increasing catalase (CAT) and glutathione peroxidase (GSH-Px) activities, elevating the glutathione/oxidized glutathione (GSH/GSSG) ratio, and decreasing the malondialdehyde (MDA) level. These findings indicated that GLP-1 could be explored as a potential antioxidant agent for application in functional foods.

## 1. Introduction

Reactive oxygen species (ROS), including hydrogen peroxide (H_2_O_2_), superoxide anion, and hydroxyl free radicals, are generated during normal cellular metabolism [1]. Under normal physiological conditions, the production and elimination of ROS are usually balanced by various antioxidant compounds and enzymes. However, under pathological conditions, ROS excessively accumulate and attack proteins, lipids, and DNA, which in turn causes oxidative damage to tissues and organs [2,3]. Such oxidative injuries promote the development of various human diseases, including cardiovascular disease, age-related disorders, metabolic disease, cancer, and other diseases [4,5].

Today, natural polysaccharides are receiving increasing attention owing to their low toxicity and diverse pharmacological activities [6]. Fungal polysaccharides are an important class of functional biomacromolecules that exist in edible and medicinal fungi [7]. *Ganoderma*, one of the most popular fungi species in China and other Asian countries, has been shown to promote health and longevity [8]. Polysaccharides are the primary bioactive components in *Ganoderma* species, and they have been demonstrated to possess various biological activities, such as immunomodulatory [9], anti-aging [10], anti-tumor [11], and antioxidant activities [8]. Chen et al. [12] isolated a water-soluble protein-bound polysaccharide with an average molecular weight of 1013 kDa from the fruiting bodies of *G. atrum* and found that the polysaccharide had strong superoxide anion and DPPH radical scavenging capacities. Tseng et al. [13] reported that polysaccharides extracted from *G. tsugae* by hot water and hot alkali possessed good antioxidant properties. Several studies have confirmed that *G. lucidum* polysaccharides can significantly increase antioxidant enzyme activities in vivo [14,15].

*Ganoderma leucocontextum* is a new species of *Ganoderma* discovered in southwestern China in 2014 [16]. Several studies have revealed that terpenoids from *G. leucocontextum* possess potential beneficial effects, including antidiabetic [17], antitumor [18], and neuroprotective activities [19]. However, the structural characteristics and antioxidant activities of polysaccharides from *G. leucocontextum* remain mostly unknown.

In the present study, two low-molecular-weight polysaccharides, GLP-1 and GLP-2, were isolated and purified from the fruiting bodies of *G. leucocontextum*. Their physicochemical properties and in vitro antioxidant activities were investigated. The chemical structure of GLP-1, which had a stronger antioxidant capacity, was further characterized. In addition, the protective effects of GLP-1 on cellular oxidative stress were evaluated. This study’s results can further clarify the structure and antioxidant properties of *G. leucocontextum* polysaccharides.

## 2. Materials and Methods

### 2.1. Chemical Reagents

Monosaccharide standards (rhamnose, ribose, fucose, arabinose, xylose, mannose, glucose, galactose), dextran standards, 1-phenyl-3-methyl-5-pyrazolone (PMP), H_2_O_2_ (3%), 2,2′-azino-bis(3-ethylbenzthiazoline-6-sulfonic acid) (ABTS), 2,4,6-tris(2-pyridyl)-s-triazine (TPTZ), 6-hydro-xy-2,5,7,8-tetramethyl-2-carboxylic acid (Trolox), ascorbic acid (Vc), *tert*-butyl hydroperoxide solution (tBHP, 70%), 2,2′-azobis(2-methylpropionamidine) dihydrochloride (AAPH), fluorescein sodium salt, and deuterium oxide (D_2_O) were purchased from Sigma-Aldrich (St. Louis, MO, USA). DEAE-Sepharose fast-flow, Sephacryl S-300 HR, and penicillin–streptomycin solution were obtained from GE Healthcare Life Science (Piscataway, NJ, USA). Dulbecco’s modified Eagle’s medium (DMEM), fetal bovine serum (FBS), Pierce bicinchoninic acid (BCA) protein assay kit, and Dulbecco’s phosphate-buffered saline (DPBS, pH 7.4) were purchased from Thermo Scientific (Rockford, IL, USA). Glucuronic acid, galacturonic acid, β-nicotinamide adenine dinucleotide (NADH), phenazine methosulfate (PMS), and nitroblue tetrazolium (NBT) were purchased from Shanghai Yuanye Bio-Technology Co., Ltd. (Shanghai, China). Lactate dehydrogenase (LDH), microscale malondialdehyde (MDA), and total glutathione (GSH)/oxidized glutathione (GSSG) assay kits were purchased from Nanjing Jiancheng Bioengineering Institute (Nanjing, China). Catalase (CAT), glutathione peroxidase (GSH-Px), reactive oxygen species, and Bradford protein assay kits were purchased from Beyotime Institute of Biotechnology (Shanghai, China). Cell counting kit-8 (CCK-8) was obtained from MedChemExpress (Monmouth Junction, NJ, USA). Congo red was purchased from Beijing Solarbio Science and Technology Co., Ltd. (Beijing, China). The ultrapure water was prepared by Milli-Q Integral 3 (Merck-Millipore, Molsheim, Alsace, France). Other reagents used were of analytical grade.

### 2.2. Extraction of Crude Polysaccharide

*G. leucocontextum* fruiting bodies cultured in 2018 were provided by Huiping Hu (Guangdong Institute of Microbiology, Guangzhou, China). The crude *G. leucocontextum* polysaccharide (CGLP) was obtained through hot-water extraction, ethanol precipitation, deproteinization, dialysis, and lyophilization according to our recent study [20].

### 2.3. Fractionation and Purification

CGLP was redissolved in ultrapure water and separated into three fractions (CGLP-1: 5–10 kDa, CGLP-2: 10–100 kDa, and CGLP-3: >100 kDa) using the cross-flow ultrafiltration membrane of 100 kDa and 10 kDa MWCO (200 cm^2^, PES; Sartorius, Göttingen, Germany). The structural characterization and immunomodulatory activity of purified CGLP-3 have been investigated in our group [20]. Therefore, CGLP-1 and CGLP-2 were poured into a DEAE-Sepharose fast-flow anion exchange column (2.6 cm × 30 cm) in this study. The column was eluted with ultrapure water and different concentrations of gradient NaCl solution (0.1–0.5 M) at a flow rate of 2 mL/min. The total carbohydrate content in each collected fraction (8 mL/tube) was determined by the phenol–sulfuric acid method. Subsequently, the fraction obtained from 0.1 M NaCl was further applied to a Sephacryl S-300 HR gel permeation column (2.6 cm × 60 cm) due to its higher yield. The column was eluted with ultrapure water at a flow rate of 1.0 mL/min, and eluates (8 mL/tube) were collected automatically and detected as described above. Two purified polysaccharide fractions (GLP-1 and GLP-2) were collected, concentrated, and lyophilized. The extraction yields of GLP-1 and GLP-2 were approximately 0.098% and 0.054% (the ratio of dry matter), respectively.

### 2.4. Components Analysis

The carbohydrate content was measured by the phenol–sulfuric acid method using glucose as a standard [21]. The protein content was estimated by Bradford’s method using bovine serum albumin as a standard [22].

### 2.5. Structural Characteristics

#### 2.5.1. Molecular Weight Detection

The molecular weights of GLP-1 and GLP-2 were measured by high-performance gel permeation chromatography (HPGPC), which was performed on a Waters ACQUITY APC system (Milford, MA, USA). The column was a serially linked combination of Waters ACQUITY APC AQ 900 and ACQUITY APC AQ 450 column (2.5 μm × 4.6 mm × 150 mm, Milford, MA, USA). Samples were eluted using NaNO_3_ (100 mM) solution at a flow rate of 0.4 mL/min. The column’s temperature was maintained at 35 °C. The molecular weights of GLP-1 and GLP-2 were calculated based on the calibration curve, which was obtained from dextran standards with different molecular weights (5.2, 11.6, 23.8, 48.6, 148, 273, 410, 668 kDa).

#### 2.5.2. Monosaccharide Composition Analysis

The monosaccharide compositions of GLP-1 and GLP-2 were analyzed by high-performance liquid chromatography (HPLC), as described in a previous study [23], but with some modifications. A polysaccharide sample (2 mg) was hydrolyzed with 2 M trifluoroacetic acid (1 mL) at 110 °C for 6 h, followed by derivatization with 0.5 M PMP. The PMP derivatives were analyzed on an Agilent 1200 Series HPLC system (G1322A Degasser, G1311A Quat Pump, G1329A ALS, G1315D DAD, Agilent Technologies, Inc., Santa Clara, CA, USA) equipped with an Eclipse XDB-C18 column (250 mm × 4.6 mm × 5 µm, Agilent, Santa Clara, CA, USA) at 30 °C. The detection wavelength was set at 250 nm, and the flow rate was 0.8 mL/min. The mobile phase was a mixture of phosphate-buffered saline (0.1 M, pH 6.5) and acetonitrile (84:16, *v*/*v*). Rhamnose, ribose, fucose, arabinose, xylose, mannose, glucose, galactose, glucuronic acid, and galacturonic acid were used as standards.

#### 2.5.3. Fourier-Transform Infrared Spectroscopy (FT-IR) Analysis

The FT-IR spectra of GPL-1 and GLP-2 were recorded at the range of 4000–400 cm^−1^ with potassium bromide pellets using a Vertex 70 spectrometer (Bruker, Karlsruhe, Germany).

#### 2.5.4. Congo Red Test

The conformational structures of GLP-1 and GLP-2 were determined using the Congo red method described by Gao et al. [20].

#### 2.5.5. X-ray Diffraction (XRD) Analysis

The X-ray diffraction patterns of GLP-1 and GLP-2 were measured using a D8 Advance X-ray diffractometer (Bruker, Karlsruhe, Germany). The 2θ angle from 5° to 90° was scanned at a rate of 10 °/min with working current and voltage set to 40 mA and 40 kV, respectively.

#### 2.5.6. Molecular Surface Morphology Analysis

Polysaccharide aqueous solution (5 µg/mL) was dropped onto the freshly stripped mica and dried in the air for 1.5 h. The atomic force microscope (AFM) (NanoMan VS, Veeco, Plainview, New York, NY, USA) was used to scan the surface topology in tapping mode.

#### 2.5.7. Methylation Analysis

GLP-1 (6 mg) was methylated, hydrolyzed, reduced, and acetylated according to a previous method [24]. The reaction product was analyzed by gas chromatography–mass spectrometry (GC–MS) on a GCMS-QP2010 system (Shimadzu, Columbia, MD, USA) equipped with an RXI-5 SIL MS column (30 m × 0.25 mm × 0.25 μm, Restek, Bellefonte, PA, USA). Temperature gradient profile was as follows: initial column temperature (120 °C) was increased to 250 °C at a rate of 3 °C/min, and maintained at 250 °C for 5 min. Helium (He) was used as the carrier gas at 1 mL/min.

#### 2.5.8. Nuclear Magnetic Resonance (NMR) Spectroscopy Analysis

Dried GLP-1 (50 mg) was completely dissolved in 0.5 mL of D_2_O. The solution was then transferred into a 5 mm NMR tube and analyzed by a Bruker AVANCE III 600 MHz spectrometer (Rheinstetten, Germany) to obtain 1D NMR (^1^H NMR, ^13^C NMR, and DEPT135) and 2D NMR (^1^H-^1^H COSY, HSQC, and HMBC).

### 2.6. Evaluation for In Vitro Antioxidant Activities

#### 2.6.1. ABTS Radical Cation Decolorization Assay

The antioxidant capacity of polysaccharide samples in the reaction with ABTS radical cation (ABTS^•+^) was determined by the method of Re et al. [25] with some modifications, applied to a 96-well microplate assay. First, ABTS^•+^ was produced by reacting 7 mM ABTS stock solution with 2.45 mM potassium persulfate (final concentration) and allowing the mixture to stand in the dark at room temperature for 16 h before use. The ABTS^•+^ solution was diluted with PBS to obtain an absorbance of 0.70 (±0.05) at 734 nm by mixing with an equal volume of ultrapure water. Then, 100 µL of appropriately diluted samples was mixed with 100 µL of ABTS^•+^ solution. The reaction mixture was incubated in the dark for 6 min at 30 °C. The absorbance at 734 nm was read using a VersaMax ELISA microplate reader (Molecular Devices, Sunnyvale, CA, USA), and Vc was used as a positive control. The ABTS^•+^ radical scavenging rate was calculated using the following formula:

ABTS^•+^ radical scavenging activity (%) = [(*A_control_* − *A_sample_*)/*A_control_*] × 100 (1)

where *A_sample_* is the absorbance of the tested sample and *A_control_* is the absorbance of the ultrapure water instead of the tested sample.

#### 2.6.2. Hydroxyl Radical Scavenging Assay

The hydroxyl radical scavenging capacity of polysaccharide samples was evaluated on a microplate analytical assay according to a previous method [26] with some modifications. First, 50 µL of ferrous sulfate (1.5 mM) and 50 µL of H_2_O_2_ (0.01%) were mixed with 100 µL of samples. Finally, 50 µL of 1,10-phenanthroline (1.5 mM) was added. The reaction mixture was then incubated in the dark for 30 min at 37 °C, and the absorbance was measured at 536 nm. Vc was used as a positive control. The hydroxyl radical scavenging rate was calculated using the following formula:

Hydroxyl radical scavenging activity (%) = (*A_sample_* − *A_control_*)/(*A_0_* − *A_control_*) × 100 (2)

where *A_sample_* is the absorbance of the tested sample, *A_control_* is the absorbance of the ultrapure water instead of the tested sample, and *A_0_* is the absorbance of the ultrapure water instead of H_2_O_2_ and the tested sample.

#### 2.6.3. Superoxide Anion Scavenging Assay

The superoxide anion scavenging activity of polysaccharide samples was evaluated on a microplate analytical assay according to the method of Li et al. [27] with some modifications. First, 100 µL of 0.1 M sodium phosphate buffer (pH 7.4) containing 375 µM NADH and 125 µM NBT was mixed with 100 µL of samples. After the addition of 16.5 µM PMS (50 µL), the mixture was incubated in the dark for 5 min at 25 °C. The absorbance was recorded at 560 nm, and Vc was used as a positive control. The superoxide anion scavenging rate was calculated using the following formula:

Superoxide anion radical scavenging activity (%) = [(*A_control_* − *A_sample_*)/*A_control_*] × 100 (3)

where *A_sample_* is the absorbance of the tested sample and *A_control_* is the absorbance of the ultrapure water instead of the tested sample.

#### 2.6.4. Ferric Reducing Antioxidant Power Assay

The ferric reducing antioxidant power (FRAP) assay was performed according to the method of Benzie and Strain [28] with slight modifications, applied to a 96-well microplate assay. The working FRAP reagent was made 1 h prior to the assay by mixing 300 mM acetate buffer pH 3.6 (1.896 g CH_3_COONa and 16 mL CH_3_COOH per liter), 10 mM TPTZ solution in 40 mM HCl, and 20 mM FeCl_3_ solution in ultrapure water in the ratio of 10:1:1. The FRAP reagent was warmed to 37 °C before the assay. Then, 150 µL of FRAP reagent was mixed with 50 µL of appropriately diluted samples or Trolox. The reaction mixture was incubated in the dark for 4 min at 37 °C, and the absorbance was measured at 593 nm. Trolox equivalents were calculated using a calibration curve prepared with Trolox (0–40 µM, final concentration). Results were expressed as µmol/g Trolox equivalent antioxidant capacity (TEAC).

#### 2.6.5. Oxygen Radical Antioxidant Capacity Assay

The oxygen radical antioxidant capacity (ORAC) assay of polysaccharide samples was performed according to a previous method [29] with slight modifications. The fluorescence measurement was performed at 37 °C on a SpectraMax i3x multi-mode microplate reader (Molecular Devices, Sunnyvale, CA, USA). The fluorescence intensity was recorded every minute for 60 min at an emission wavelength of 520 nm and an excitation wavelength of 485 nm. Trolox equivalents were calculated using a calibration curve prepared with Trolox (0–4 µM, final concentration). The final ORAC values were expressed as µmol/g TEAC.

### 2.7. Intracellular Antioxidant Activities of GLP-1

#### 2.7.1. Cell Culture

Mouse embryonic fibroblast cells (NIH3T3) were obtained from the Chinese Academy of Sciences (Shanghai, China) and cultured in DMEM with 10% FBS and 1% penicillin–streptomycin solution. The cells were incubated in a humidified atmosphere at 37 °C with 5% CO_2_.

#### 2.7.2. Measurement of Cell Viability and LDH Release

Before the investigation of the protective effect of GLP-1 on tBHP-induced cellular oxidative damage, NIH3T3 cells were treated with various tBHP concentrations (50, 75, 100, 125, and 150 μM) for 24 h to confirm an appropriate concentration in this cell model. Briefly, NIH3T3 cells were seeded on a 96-well plate at a density of 1 × 10^4^ cells/well and incubated for 24 h at 37 °C in 5% CO_2_. Subsequently, the cells were treated with different concentrations of GLP-1 (0.5, 1, and 2 mg/mL) or Trolox (80 µM) and co-cultured with 100 µM of tBHP. The blank control group and model group were treated with DMEM and 100 µM of tBHP, respectively. After 24 h of treatment, the cell culture supernatants were collected for LDH release assay according to the manufacturer’s instructions. Cell viability was measured with 200 µL of serum-free DMEM containing CCK-8 solution (5 µL). The plate was incubated for another 2 h at 37 °C and the absorbance was recorded at 450 nm. The cell viability was expressed as the percentage of the blank control group.

#### 2.7.3. Determination of Intracellular Reactive Oxygen Species

The intracellular reactive oxygen species (ROS) of NIH3T3 cells were determined using an ROS assay kit. First, NIH3T3 cells (5 × 10^4^ cells/well) were seeded on a 24-well plate and incubated for 24 h at 37 °C in 5% CO_2_. After 6 h of various treatments as described above, the medium was removed, and 0.5 mL of serum-free DMEM containing DCFH-DA (10 µM) was added into each well. The plate was transferred to the incubator for 30 min, and then washed with DPBS three times. Fluorescence images were captured using an EVOS FL Auto 2 microscope (Thermo Fisher Scientific, Bothell, WA, USA). The fluorescence intensity of each image was quantified by ImageJ software.

#### 2.7.4. Determination of MDA, GSH, GSSG, CAT, and GSH-Px Levels

First, NIH3T3 cells (6 × 10^5^ cells/well) were seeded on 60 mm^2^ culture dishes and incubated for 24 h at 37 °C in 5% CO_2_. After 24 h of different treatments as described above, the cells were collected and the cell lysate supernatant was used in the next analysis. The protein concentrations were quantified by the BCA assay kit. The contents of MDA, GSH, and GSSG were measured with microscale MDA and total GSH/GSSG assay kits. The activities of CAT and GSH-Px were analyzed by CAT and GSH-Px assay kits. These experiments were conducted according to the manufacturer’s instructions.

### 2.8. Statistical Analysis

The results were presented as means ± standard deviation (S.D.). The statistical significance of difference was evaluated using one-way analysis of variance (ANOVA) followed by Fisher’s least significant difference (LSD) test using SAS 9.2 software. Origin 9.2 software was used for illustration.

## 3. Results and Discussion

### 3.1. Purification, Component Analysis and Molecular Weights of GLP-1 and GLP-2

**Figure 1:**
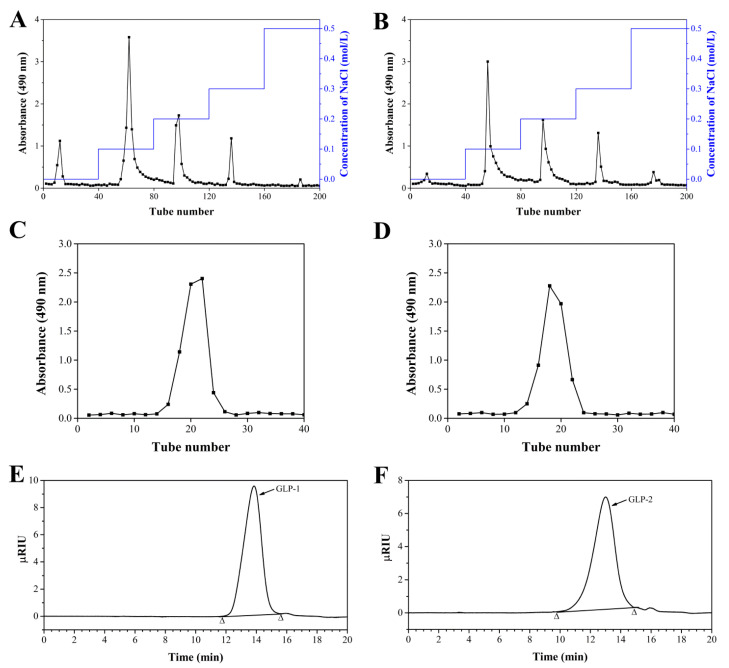
Stepwise elution profiles of CGLP-1 (**A**) and CGLP-2 (**B**) on DEAE-Sepharose fast-flow column. Elution profiles of the 0.1 M NaCl fraction obtained from CGLP-1 (**C**) and CGLP-2 (**D**) on Sephacryl S-300 HR column. HPGPC graphs of GLP-1 (**E**) and GLP-2 (**F**).

Using an ultrafiltration instrument, the water-soluble CGLP was separated into three fractions: 5–10 kDa (CGLP-1), 10–100 kDa (CGLP-2), and >100 kDa (CGLP-3). The structural characteristics and immunomodulatory activity of purified CGLP-3 have been investigated in our previous study [20]. In the current study, to obtain homogenized polysaccharides, both CGLP-1 and CGLP-2 were subjected to a DEAE-Sepharose fast-flow column and eluted with ultrapure water and 0.1–0.5 M NaCl (Figure 1A,B). The fractions obtained from 0.1 M NaCl, which were the highest yield, were further purified by a Sephacryl S-300 HR column (Figure 1C,D). The obtained GLP-1 and GLP-2 exhibited a single peak in the GPC chromatogram (Figure 1E,F).

The chemical compositions and molecular weights of GLP-1 and GLP-2 are presented in Table 1. The total carbohydrate contents of GLP-1 and GLP-2 were 73.36% and 72.45%, respectively. Both GLP-1 (0.03%) and GLP-2 (0.08%) contained very low protein, suggesting that protein was almost entirely removed by the Sevage reagent. According to the HPGPC analysis, the weight-average molecular weight (Mw) of GLP-1 was calculated to be 6.31 kDa, while that of GLP-2 was calculated to be 14.07 kDa according to the calibration curve for the standard. The polydispersity indexes (Mw/weight-average molecular weight (Mn)) of GLP-1 and GLP-2 were 1.21 and 1.40, respectively, indicating that both GLP-1 and GLP-2 had a relatively homogeneous molecular weight.

### 3.2. Monosaccharide Compositions of GLP-1 and GLP-2

**Figure 2:**
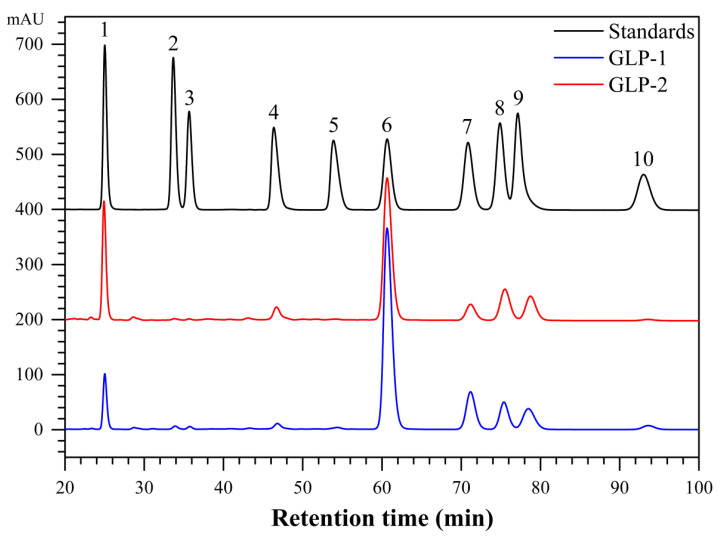
Monosaccharide compositions of GLP-1 and GLP-2 analyzed by HPLC (1—mannose, 2—ribose, 3—rhamnose, 4—glucuronic acid, 5—galacturonic acid, 6—glucose, 7—galactose, 8—xylose, 9—arabinose, and 10—fucose).

The monosaccharide compositions of GLP-1 and GLP-2 were analyzed by the PMP–HPLC method. According to the monosaccharide standards, GLP-1 mainly comprised mannose, glucose, galactose, xylose, and arabinose in a molar ratio of 7.02:60.85:12.00:8.58:7.51 along with small amounts of ribose, rhamnose, glucuronic acid, and fucose in a molar ratio of 0.58:0.55:1.10:1.80. The polysaccharide GLP-2 contained mannose, glucuronic acid, glucose, galactose, xylose, and arabinose in a molar ratio of 17.95:3.24:50.75:6.08:12.79:9.19 (Figure 2). These results indicated that glucose was the predominant monosaccharide constituting the backbones of GLP-1 and GLP-2, which is similar to the case of the polysaccharides extracted from *G. atrum* [12] and *G. lucidum* [30].

### 3.3. FT-IR Spectra of GLP-1 and GLP-2

The FT-IR spectra showed that both GLP-1 and GLP-2 contained the typical absorption peaks of polysaccharides (Figure S1). The characteristic peak at 3392.4 cm^−1^ was due to the O–H stretching vibration, and the peak at 2925.6 cm^−1^ was due to the C–H stretching vibration [31,32]. The absorption peak around 1726.1 cm^−1^ was attributed to the stretching vibrations of carboxylic groups [33]. The polysaccharide GLP-1 had a weaker absorption peak at 1726.1 cm^−1^ than GLP-2, which might be due to the lower uronic acid content of GLP-1 (described above). The absorption peak at 1645.4 cm^−1^ for GLP-1 and that at 1658.2 cm^−1^ for GLP-2 indicated the presence of associated water [34,35]. The bands in the range of 1200–1500 cm^−1^ probably corresponded to the deformation vibrations of C–H and bending vibrations of C–OH [36]. The intense bands at 1154.7, 1076.5, and 1040.0 cm^−1^ indicated the pyranose form of glucosyl residues [37,38]. In addition, the characteristic absorptions at 835.4 and 900.3 cm^−1^ suggested the presence of *α*- and *β*-type glycosidic linkage, respectively [6,39].

### 3.4. Chain Conformation and Crystalline Characteristics of GLP-1 and GLP-2

**Figure 3:**
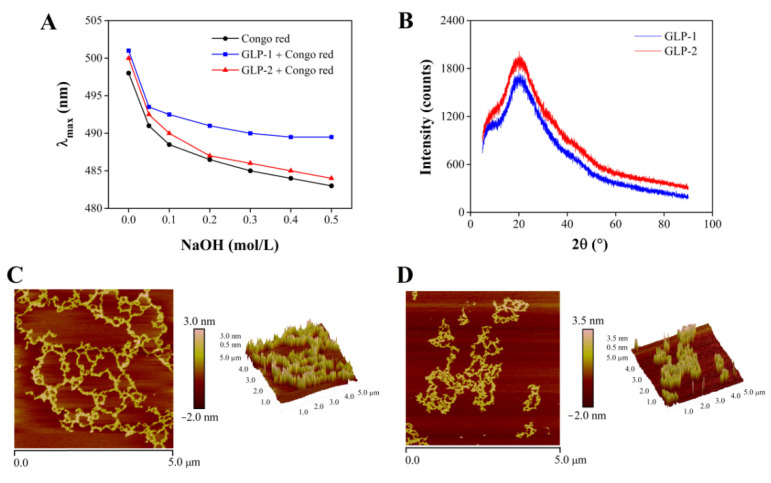
Maximum absorption wavelength of Congo red–polysaccharide complex and Congo red at different NaOH concentrations (**A**). XRD analysis results of GLP-1 and GLP-2 (**B**). AFM planar and cubic images of GLP-1 (scan size: 5 μm) (**C**). AFM planar and cubic images of GLP-2 (scan size: 5 μm) (**D**).

Polysaccharides with a triple-helix structure can form complexes with Congo red, so that the λ_max_ of the complex will undergo a bathochromic shift in comparison to Congo red [40]. The result of the Congo red experiment is shown in Figure 3A. No redshifts of the λ_max_ were observed in the concentration range of 0.05–0.5 M, indicating that no triple-helix structure existed in GLP-1 and GLP-2. A previous study also reported that no triple-helix conformation existed in polysaccharides extracted from *G. lucidum* by ultrasound and hot water [8].

The crystalline structures of GLP-1 and GLP-2 were examined by XRD. As shown in Figure 3B, the diffraction curves had amorphous peak regions at the angles (2θ) around 20° and contained no sharp peaks, suggesting that GLP-1 and GLP-2 were low-crystallinity amorphous polymers [41]. Similar diffraction peaks at about 20° were also observed in the polysaccharides from *G. lucidum* [42] and *Bletilla striata* [43].

### 3.5. Morphological Properties of GLP-1 and GLP-2

AFM has been widely used to characterize the morphological properties of biological macromolecules including polysaccharides [44]. The planar and three-dimensional structures of GLP-1 and GLP-2 are shown in Figure 3C,D. The network structures of GLP-1 and GLP-2 in aqueous solution were observed. The height and width of the chain were in the ranges of 0.5–3.5 nm and 70–240 nm, respectively. The theoretical diameter of a single polysaccharide chain is generally 0.1–1.0 nm [45]. These results suggested that the polysaccharide units could aggregate in aqueous solution, a behavior also exhibited by polysaccharides from *Lentinus edodes* [46]. Giannotti et al. [47] have demonstrated the hydrogen-bonded water-bridged nature of the network structure of polysaccharide chains. Li et al. [48] pointed out that hydrogen bonding triggered the molecular aggregation of polysaccharides because the hydroxyl groups on the chains provided strong inter- and intra-molecular interactions with each other or water molecules. Therefore, the network structures of GLP-1 and GLP-2 were probably due to hydrogen bonding interactions.

### 3.6. Antioxidant Activities of GLP-1 and GLP-2 In Vitro

Nowadays, many antioxidant methods are widely used to screen antioxidant compounds, but various analytical methods have different mechanisms and suitability. Therefore, a single method cannot accurately and quantitatively assess the antioxidant capacity, and two or more methods with different mechanisms of antioxidant action are suggested [49,50]. In this study, ABTS, hydroxyl radical, superoxide anion radical, FRAP, and ORAC assays were carried out to compare the antioxidant activities of GLP-1 and GLP-2.

**Figure 4:**
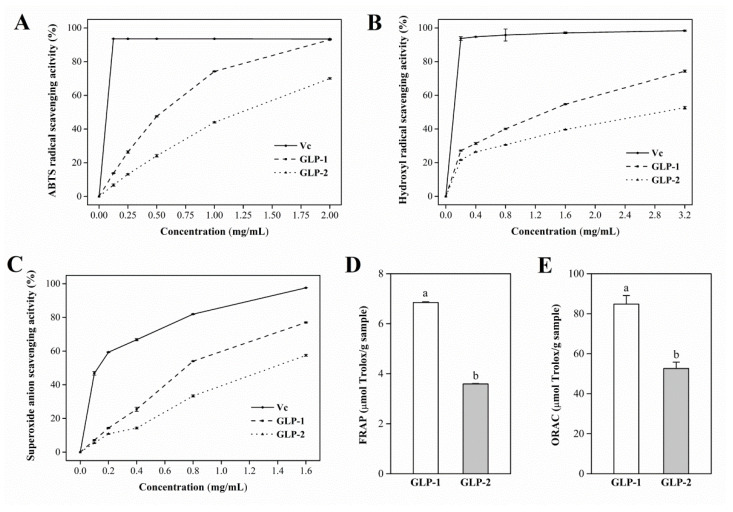
Antioxidant activities of GLP-1 and GLP-2 in five different assays: scavenging capacity of ABTS radical (**A**); scavenging capacity of hydroxyl radical (**B**); scavenging capacity of superoxide anion radical (**C**); TEAC values for FRAP assay (**D**); TEAC values for ORAC assay (**E**). Data are presented as mean ± S.D. Values with different letters (a,b) denote the significant differences (*p* < 0.05).

As shown in Figure 4, both GLP-1 and GLP-2 exhibited obvious ABTS, hydroxyl radical, and superoxide anion radical scavenging activities in a concentration-dependent manner. Moreover, the IC_50_ values of GLP-1 for ABTS, hydroxyl radical, and superoxide anion radical were 0.56 mg/mL, 1.32 mg/mL, and 0.76 mg/mL, respectively, which were lower than those of GLP-2 (1.18 mg/mL, 2.78 mg/mL, and 1.34 mg/mL). However, the radical scavenging abilities of Vc were relatively higher than those of GLP-1 and GLP-2. For FRAP and ORAC assays (Figure 4D,E), the TEAC values of GLP-1 were 6.85 µmol/g and 84.8 µmol/g, respectively, which were 2.77 and 1.61 times larger than those of GLP-2 (3.59 µmol/g and 52.6 µmol/g, respectively). The TEAC values of GLP-1 and GLP-2 from the FRAP assay were lower than those from the ORAC assay. On one hand, this was due to the different reaction mechanisms of these two methods. On the other hand, both GLP-1 and GLP-2 exhibited weak reducing power, which was consistent with the results from a previous study [51]. The results of five antioxidant methods consistently indicated that GLP-1 had better antioxidant activity than GLP-2.

Although many studies have demonstrated that polysaccharides possess antioxidant activities, the underlying mechanism is still not fully understood. It has been reported that the molecular weight and uronic acid contents of polysaccharides are two important parameters related to antioxidant abilities [52,53]. The presence of uronic acid groups in the polysaccharides can activate the hydrogen atom of the anomeric carbon [52]. In several studies, polysaccharides with higher uronic acid contents were found to generally have stronger antioxidant properties [34,54]. Our results showed that GLP-1 exhibited better antioxidant capacity despite the fact that the uronic acid content in GLP-1 was lower than that in GLP-2. This may be correlated to the different molecular weights between GLP-1 and GLP-2. In some previous studies, polysaccharides with relatively larger molecular weights showed better antioxidant efficiency [55,56]. Nevertheless, Cai et al. [53] found that a low-Mw polysaccharide from *Sophorae tonkinensis* Radix was more effective in free-radical scavenging and Fe^2+^ chelating. Liu et al. [51] reported that a low-Mw polysaccharide (5.2 kDa) from *G. lucidum* displayed better antioxidant activity than a high-Mw polysaccharide (15.4 kDa), which is consistent with our result. Therefore, the stronger antioxidant properties of GLP-1 could be partly due to its relatively lower molecular weight.

### 3.7. Protective Effects of GLP-1 on tBHP-Induced Oxidative Damage in NIH3T3 Cells

**Figure 5:**
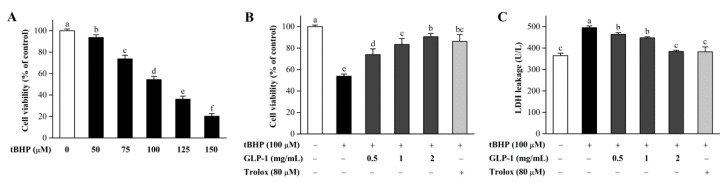
The viability of NIH3T3 cells treated with various tBHP concentrations (**A**). The viability (**B**) and LDH leakage (**C**) of NIH3T3 cells treated with different GLP-1 concentrations or Trolox and co-cultured with 100 µM of tBHP. Data are presented as mean ± S.D. Values with different letters (a–f) denote the significant differences (*p* < 0.05).

To confirm an appropriate concentration in the cell model, NIH3T3 cells were treated with different tBHP concentrations. As shown in Figure 5A, after treatment with 100 μM of tBHP, the viability of NIH3T3 cells significantly decreased to 54.4%. Therefore, tBHP at a concentration of 100 μM was used to induce oxidative damage in the subsequent experiments.

As shown in Figure 5B, compared with the model group, treatment with various GLP-1 concentrations (0.5 mg/mL, 1 mg/mL, and 2 mg/mL) increased cell viability in a dose-dependent manner. The viability of NIH3T3 cells treated with 2 mg/mL of GLP-1 was significantly increased to 90.5%. As a stable cytoplasmic enzyme, LDH was a vital marker to evaluate cellular injury [34]. As illustrated in Figure 5C, LDH leakage markedly increased after the treatment with 100 μM tBHP. However, GLP-1 inhibited the LDH release in a dose-dependent manner. Moreover, 2 mg/mL of GLP-1 reduced the LDH levels to a normal level. The protective effects of GLP-1 at 2 mg/mL were comparable to those of Trolox (80 μM). These results indicated that GLP-1 could prevent tBHP-induced oxidative damage in NIH3T3 cells.

**Figure 6:**
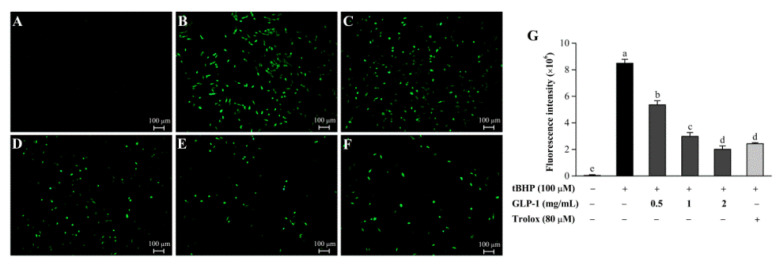
Effect of GLP-1 on intracellular ROS level: blank control group (**A**); cells treated with 100 μM tBHP (**B**); 100 μM tBHP + 0.5 mg/mL GLP-1 (**C**); 100 μM tBHP + 1 mg/mL GLP-1 (**D**); 100 μM tBHP + 2 mg/mL GLP-1 (**E**); 100 μM tBHP + 80 μM Trolox (**F**). Fluorescence intensity analysis (**G**). Data are presented as mean ± S.D. Values with different letters (a–d) denote the significant differences (*p* < 0.05).

Excessive intracellular ROS can cause oxidative stress through the oxidation of biomolecules in cells and tissues [57]. Antioxidants can protect cells from oxidative damage by reducing the ROS levels [58]. To explore the underlying mechanisms by which GLP-1 protected against tBHP, the effect of GLP-1 on intracellular ROS levels was investigated. As presented in Figure 6, compared with the blank control group, a significant increase in ROS production was observed in NIH3T3 cells after tBHP treatment for 6 h. However, the GLP-1-treated groups significantly reduced ROS generation compared with the tBHP-induced group. These results suggested that GLP-1 could protect NIH3T3 cells from tBHP-induced oxidative damage by inhibiting intracellular ROS production.

**Figure 7:**
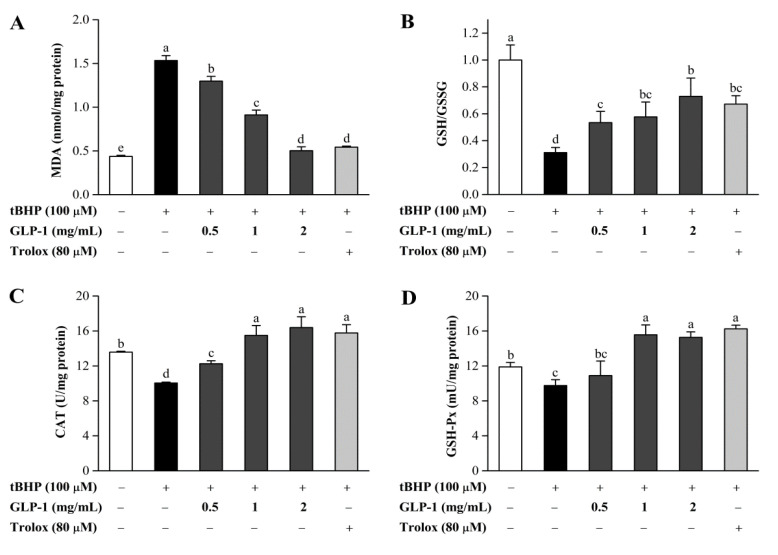
Effects of GLP-1 on MDA level (**A**), GSH/GSSG ratio (**B**), CAT (**C**), and GSH-Px (**D**) activities in tBHP-treated NIH3T3 cells. Data are presented as mean ± S.D. Values with different letters (a–d) denote the significant differences (*p* < 0.05).

Overproduced ROS can react with the double bonds of polyunsaturated fatty acids in cell membranes and consequently generate lipid hydroperoxides. MDA, a marker of lipid peroxidation, has been reported to accumulate in various diseases related to free radical damage [59]. As shown in Figure 7A, tBHP-treated NIH3T3 cells increased the MDA level by a factor of 2.5. The polysaccharide GLP-1 significantly suppressed the MDA accumulation in a dose-dependent manner, and the inhibitory effect of 2 mg/mL GLP-1 was comparable to that of 80 μM Trolox. Glutathione, a nonenzymatic antioxidant in the cells, plays a crucial role in the antioxidant defense system. A too-high level of GSSG may damage many enzymes; thus, the GSH/GSSG ratio is a good index of oxidative damage in cells [2]. As can be seen in Figure 7B, the GSH/GSSG ratio markedly reduced in NIH3T3 cells induced by tBHP. However, GLP-1 treatment significantly inhibited the decrease in the GSH/GSSG ratio of the tBHP-treated group.

The antioxidant enzyme system plays an important role in the protection against oxidative stress. Catalase and GSH-Px are included in this system, and their activities have been widely used as important antioxidant biomarkers [7,60]. Catalase converts H_2_O_2_ to H_2_O, and GSH-Px participates in catalyzing the reaction of hydroperoxides, which requires GSH as the electron donor. Therefore, their activities are crucial for maintaining the steady-state concentration of H_2_O_2_ and the control level of lipid hydroperoxides [61,62]. As shown in Figure 7C,D, compared with the blank control, NIH3T3 cells exposed to tBHP featured a significant decrease in CAT and GSH-Px activities. Nevertheless, GLP-1 treatment significantly elevated CAT and GSH-Px activities in comparison to the tBHP-treated group. Additionally, GLP-1 at higher concentrations (1 mg/mL and 2 mg/mL) markedly enhanced CAT and GSH-Px activities compared with those of the untreated cells. These findings indicated that GLP-1 might protect NIH3T3 cells against oxidative damage through the enzymatic mechanism.

### 3.8. Linkage Features of GLP-1

Methylation is effectively used to determine the glycosidic linkage pattern of polysaccharides. The linkage types in GLP-1, obtained based on the literature data [24,63,64] and a mass spectrum analysis conducted in this study, are presented in Table 2. The polysaccharide GLP-1 contained five terminal residues, five linear glycosidic residues, and four branching glycosidic residues. The dominant residues including Glc*p*-(1→, →3)-Glc*p*-(1→, →4)-Glc*p*-(1→, →6)-Glc*p*-(1→, →6)-Gal*p*-(1→, and →4,6)-Glc*p*-(1→ accounted for 11.29%, 12.75%, 25.89%, 10.14%, 11.97%, and 8.69%, respectively. The total content of non-reducing terminals agreed with that of branching glycosidic residues, indicating that GLP-1 was a branched polysaccharide.

### 3.9. NMR-Derived Structural Characteristics of GLP-1

**Figure 8:**
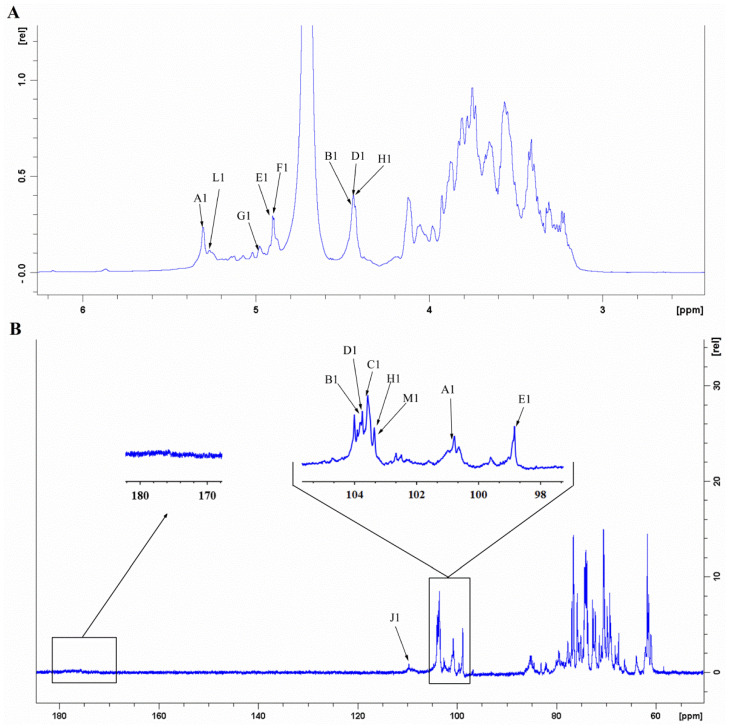
^1^H NMR (**A**) and ^13^C NMR (**B**) spectra of GLP-1.

NMR spectroscopy was used to obtain the detailed structural information of GLP-1. The signals at 5.32, 4.45, 4.45, 4.91, 4.89, 5.00, 4.44, and 5.26 ppm in the ^1^H NMR spectrum corresponded to H-1 of **A**, **B**, **D**, **E**, **F**, **G**, **H**, and **L** residues, respectively (Figure 8A). The main anomeric carbon signals observed at 100.66, 103.63, 103.48, 103.63, 98.79, 103.36, 108.95, and 103.32 ppm in the ^13^C NMR spectrum corresponded to the C-1 of **A**, **B**, **C**, **D**, **E**, **H**, **J**, and **M** residues, respectively (Figure 8B). The inverted signals at 61.43, 60.96, 61.74, 67.37, and 62.30 ppm in the DEPT135 spectrum corresponded to the C-6 of **A**, **B**, **C**, **E**, and **L** residues, respectively (Figure S2). The signals at 16.57/1.15 ppm might be due to the methyl of Rha residues (**K**).

**Figure 9:**
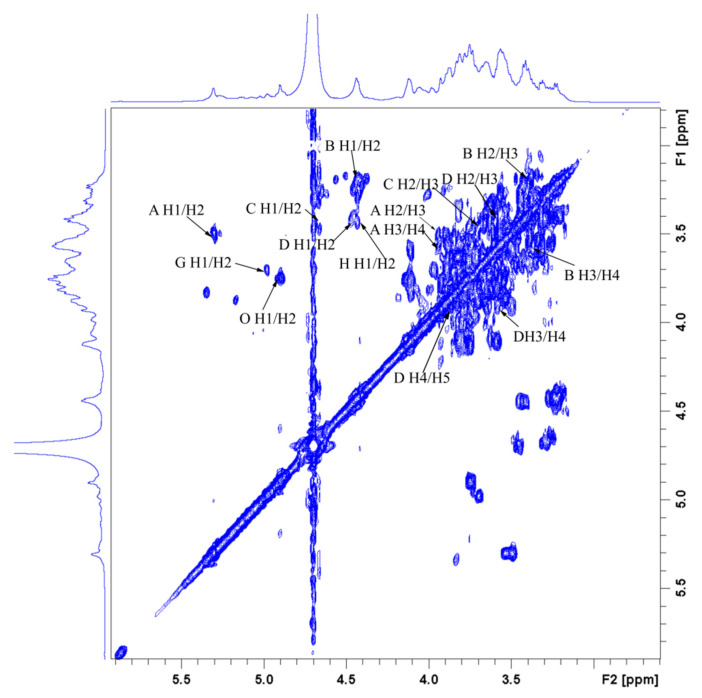
^1^H-^1^H COSY spectrum of GLP-1.

**Figure 10:**
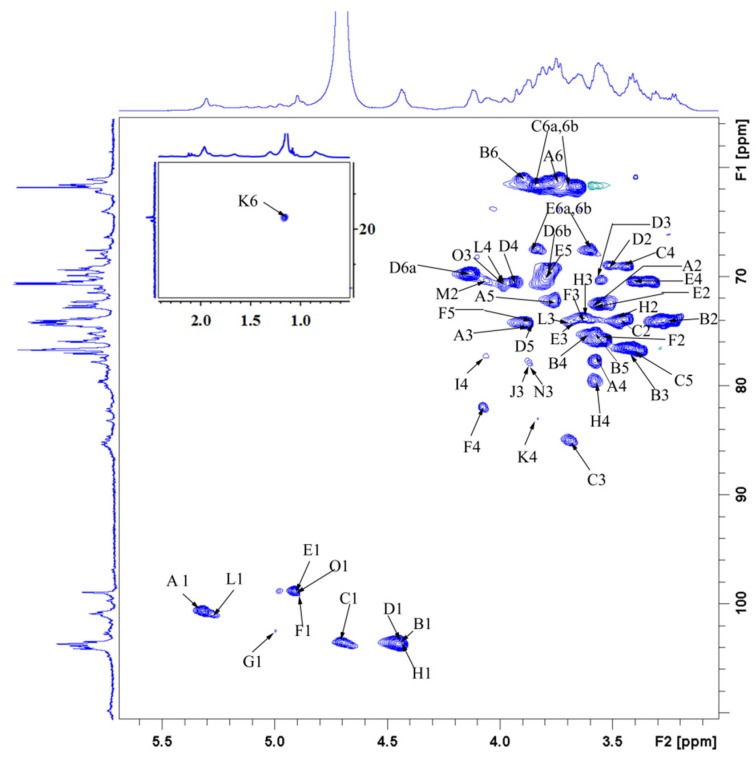
HSQC spectrum of GLP-1.

The assignations of residue signals in ^1^H and ^13^C NMR spectra were further analyzed by the ^1^H-^1^H COSY and HSQC spectra. The residue **A**, namely →4)-*α*-D-Glc*p*-(1→, was taken as an example in this section. The cross-peaks 5.32/3.55 and 3.55/3.88 ppm were detected in the ^1^H-^1^H COSY spectrum (Figure 9). Given that the signal at 5.32 ppm corresponded to the H-1 of residue **A**, 3.55 and 3.88 ppm were attributed to the H-2 and H-3 of residue **A**, respectively. Similarly, the signals at 3.58, 3.76, and 3.77 ppm were assigned to the H-4, H-5, and H-6 of residue **A**. From the HSQC spectrum (Figure 10), the strong cross-peak H/C (5.32/100.66 ppm) revealed close connectivity between H-1 and C-1. The carbon peaks of C-2 (72.53 ppm), C-3 (74.25 ppm), C-4 (77.69 ppm), C-5 (72.22 ppm), and C-6 (61.43 ppm) were also found in the HSQC spectrum. Based on the above-mentioned analogy and the literature data, the C/H chemical shifts of all residues were confirmed and are presented in Table 3 [24,34,64,65,66,67].

**Figure 11:**
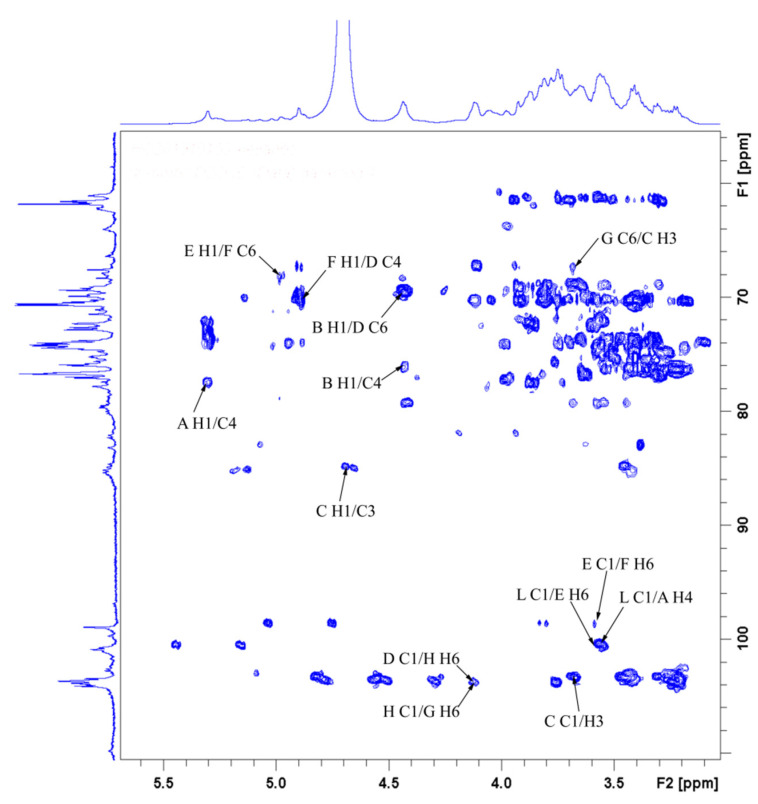
HMBC spectrum of GLP-1.

The linkage sequence and sites of glucosyl moieties were determined by the HMBC spectrum (Figure 11). Residue **C** C-1 was related to residue **B** H-4 and **C** H-3, and residue **B** C-4 was related to residue **B** H-1. Hence, the following connectivity was established: →3)-*β*-D-Glc*p*-(1→3)-*β*-D-Glc*p*-(1→4)-*β*-D-Glc*p*-(1→4)-*β*-D-Glc*p*-(1→. Inter-residual correlations were observed between residue **H** C-1 and **G** H-6, and **D** C-1 and **H** H-6. Thus, the sequence for residues **D**, **G**, and **H** was established as follows: →6)-*β*-D-Gal*p*-(1→4,6)-*β*-D-Gal*p*-(1→2,6)-*β*-D-Gal*p*-(1→. In addition, residue **D** C-6 was related to residue **B** H-1, and residue **G** C-1 was related to residue **C** H-3. Based on the above results, the backbone of GLP-1 was confirmed by the linkages of residues **B**, **C**, **D**, **G**, and **H**.

**Figure 12:**
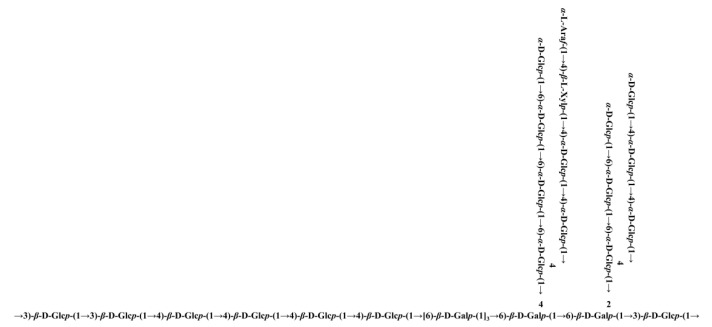
Putative structure of GLP-1.

Moreover, contacts were observed between residue **L** C-1 and **A** H-4, and **A** C-4 and **A** H-1, suggesting the presence of *α*-D-Glc*p*-(1→4)-*α*-D-Glc*p*-(1→4)-*α*-D-Glc*p*-(1→. Additionally, residue **I** C-4 correlated with residue **J** H-1, and residue **A** C-4 correlated with residue **I** H-1, which proved the existence of *α*-L-Ara*f*-(1→4)-*β*-L-Xyl*p*-(1→4)-*α*-D-Glc*p*-(1→. Inter-residual correlations were also found between residue **L** C-1 and **E** H-6, and residue **F** C-6 and **E** H-1. Hence, the following sequences were obtained: *α*-D-Glc*p*-(1→6)-*α*-D-Glc*p*-(1→4,6)-*α*-D-Glc*p*-(1→. Again, residue **D** C-4 had inter-residual contacts from residue **F** H-1, indicating the presence of →4,6)-*α*-D-Glc*p*-(1→4,6)-*β*-D-Gal*p*-(1→. Based on the monosaccharide composition analysis, methylation analysis, and NMR spectroscopy, the probable preliminary structure of GLP-1 is shown in Figure 12.

## 4. Conclusions

In this study, two low-molecular-weight polysaccharides, GLP-1 and GLP-2, were isolated and purified from *G. leucocontextum*, and their physicochemical properties and antioxidant activities were compared. The results showed that GLP-1 and GLP-2 had similar monosaccharide compositions, chain conformation, crystal structure, and molecular surface morphology, with Mw of 6.31 and 14.07 kDa, respectively. The characteristic absorption peaks of polysaccharides were observed in the FT-IR spectra of GLP-1 and GLP-2. Moreover, GLP-1, with a lower Mw than GLP-2, possessed better antioxidant capacities than GLP-2 in five different assays in vitro. Methylation analysis and NMR spectroscopy revealed that GLP-1 contained 14 kinds of linkage types. In addition, GLP-1 could inhibit ROS production and MDA accumulation in NIH3T3 cells induced by tBHP by elevating the GSH/GSSG ratio and CAT and GSH-Px activities. Our results elucidated the elaborate structure of GLP-1 and demonstrated its in vitro antioxidant activities through chemical methods and a cellular model. However, further investigation of the antioxidant property of GLP-1 in vivo is needed.

## Figures and Tables

**Table 1:** Chemical compositions and molecular weights of GLP-1 and GLP-2.

Sample	GLP-1	GLP-2
Carbohydrate (wt%)	73.36 ± 1.35	72.45 ± 0.49
Protein (wt%)	0.03 ± 0.01	0.08 ± 0.01
Mw (kDa)	6.31	14.07
Mn (kDa)	5.22	10.06
Mw/Mn	1.21	1.40

**Table 2:** Methylation analysis of GLP-1 by GC–MS.

Methylated Sugar	Linkage Pattern	Molar Ratios	Rt	Mass Fragments (*m*/*z*)
2,3,5-Me_3_-Ara*f*	T-Ara*f*-(1→	3.28	9.35	43,71,87,101,117,129,145,161
2,3,4-Me_3_-Ara*p*	T-Ara*p*-(1→	1.09	10.78	43,71,87,101,117,129,131,161
2,3,4-Me_3_-Fuc*p*	T-Fuc*p*-(1→	0.88	11.83	43,59,72,89,101,115,117,131,175
2-Me_1_-Rha*p*	→3,4)-Rha*p*-(1→	1.49	13.00	43,87,99,113,117,129,141,159,173
2,3-Me_2_-Xyl*p*	→4)-Xyl*p*-(1→	3.25	14.69	43,71,87,99,101,117,129,161,189
2,3,4,6-Me_4_-Glc*p*	T-Glc*p*-(1→	11.29	16.23	43,71,87,101,117,129,145,161,205
2,3,4,6-Me_4_-Man*p*	T-Man*p*-(1→	1.46	16.45	43,71,87,101,117,129,145,161,205
2,4,6-Me_3_-Glc*p*	→3)-Glc*p*-(1→	12.75	20.84	43,71,85,87,99,101,117,129,161
2,3,6-Me_3_-Glc*p*	→4)-Glc*p*-(1→	25.89	21.48	43,87,99,101,113,117,129,131,161,173,233
2,3,4-Me_3_-Glc*p*	→6)-Glc*p*-(1→	10.14	22.40	43,87,99,101,117,129,161,189,233
2,3,4-Me_3_-Gal*p*	→6)-Gal*p*-(1→	11.97	24.35	43,87,99,101,117,129,161,189,233
2,3-Me_2_-Gal*p*	→4,6)-Gal*p*-(1→	3.73	27.29	43,71,85,87,99,101,117,127,159,161,201
2,3-Me_2_-Glc*p*	→4,6)-Glc*p*-(1→	8.69	27.63	43,71,85,87,99,101,117,127,159,161,201
3,4-Me_2_-Gal*p*	→2,6)-Gal*p*-(1→	4.09	29.22	43,71,87,99,129,189

**Table 3:** ^13^C and ^1^H NMR chemical shifts (ppm) for GLP-1.

Glycosyl Residues	H1	H2	H3	H4	H5	H6a	H6b
	C1	C2	C3	C4	C5	C6	
→4)-*α*-D-Glc*p*-(1→	5.32	3.55	3.88	3.58	3.76	3.77	ns
**A**	100.66	72.53	74.25	77.69	72.22	61.43	
→4)-*β*-D-Glc*p*-(1→	4.45	3.24	3.42	3.58	3.54	3.90	
**B**	103.63	73.93	76.44	75.19	75.81	60.96	
→3)-*β*-D-Glc*p*-(1→	4.70	3.45	3.69	3.43	3.42	3.83	3.66
**C**	103.48	73.93	85.03	69.09	76.44	61.74	
→6)-*β*-D-Gal*p*-(1→	4.45	3.43	3.55	3.94	3.88	4.14	3.78
**D**	103.63	69.09	70.18	70.34	74.25	69.71	
→6)-*α*-D-Glc*p*-(1→	4.91	3.55	3.69	3.39	3.78	3.83	3.60
**E**	98.79	72.53	74.09	70.34	69.71	67.37	
→4,6)-*α*-D-Glc*p*-(1→	4.89	3.49	3.65	4.08	3.85	4.20	3.58
**F**	99.87	75.73	74.02	81.91	74.60	68.31	
→2,6)-*β*-D-Gal*p*-(1→	5.00	3.73	ns	ns	ns	3.91	4.12
**G**	102.38	78.94	ns	ns	ns	66.12	
→4,6)-*β*-D-Gal*p*-(1→	4.44	3.45	3.67	3.59	ns	3.90	4.13
**H**	103.36	73.76	73.75	79.71	ns	67.06	
→4)-*β*-L-Xyl*p*-(1→	5.08	3.63	3.99	4.07	3.65	4.03	
**I**	102.50	73.50	74.50	77.20	63.80		
*α*-L-Ara*f*-(1→	5.20	4.27	3.87	4.14	3.75	3.66	
**J**	108.95	82.65	77.97	85.24	62.59		
→3,4)-*α*-L-Rha*p*-(1→	4.96	3.91	3.69	3.83	3.86	1.15	
**K**	103.16	72.37	79.09	83.00	75.81	16.57	
*α*-D-Glc*p*-1→	5.26	3.60	3.70	3.95	3.99	3.60	3.82
**L**	101.33	71.94	74.03	70.61	69.25	62.30	
*α*-D-Man*p*-1→	5.03	4.06	3.62	3.51	3.76	3.83	3.66
**M**	103.32	70.34	70.50	68.93	76.28	61.74	
*α*-L-Ara*p*-(1→	5.14	4.22	3.87	4.04	3.29	3.74	
**N**	109.57	85.35	77.84	85.19	63.93		
*α*-L-Fuc*p*-1→	4.89	3.75	3.99	3.67	4.11	1.15	
**O**	98.79	73.26	70.65	76.28	68.15	16.57	

## Data Availability

Data is contained within the article or supplementary material.

## Supplementary Materials

The following are available online at https://www.mdpi.com/article/10.3390/antiox10071145/s1, Figure S1: FT-IR spectra of GLP-1 and GLP-2, Figure S2: DEPT135 spectrum of GLP-1.

## References

[1] Dikalov S.I., Harrison D.G. (2014). Methods for detection of mitochondrial and cellular reactive oxygen species. Antioxid. Redox Sign..

[2] Valko M., Leibfritz D., Moncol J., Cronin M.T.D., Mazur M., Telser J. (2007). Free radicals and antioxidants in normal physiological functions and human disease. Int. J. Biochem. Cell Biol..

[3] Rani V., Deep G., Singh R.K., Palle K., Yadav U.C.S. (2016). Oxidative stress and metabolic disorders: Pathogenesis and therapeutic strategies. Life Sci..

[4] Valko M., Jomova K., Rhodes C.J., Kuca K., Musilek K. (2016). Redox- and non-redox-metal-induced formation of free radicals and their role in human disease. Arch. Toxicol..

[5] Poprac P., Jomova K., Simunkova M., Kollar V., Rhodes C.J., Valko M. (2017). Targeting free radicals in oxidative stress-related human diseases. Trends Pharmacol. Sci..

[6] Zhou Y., Ma W., Wang L., Sun W., Li M., Zhang W., Liu Y., Song X., Fan Y. (2019). Characterization and antioxidant activity of the oligo-maltose fraction from Polygonum Cillinerve. Carbohyd. Polym..

[7] Yang W., Wang L., Gong L., Lu Y., Pan W., Wang Y., Zhang W., Chen Y. (2018). Structural characterization and antioxidant activities of a novel polysaccharide fraction from the fruiting bodies of *Craterellus cornucopioides*. Int. J. Biol. Macromol..

[8] Kang Q., Chen S., Li S., Wang B., Liu X., Hao L., Lu J. (2019). Comparison on characterization and antioxidant activity of polysaccharides from *Ganoderma lucidum* by ultrasound and conventional extraction. Int. J. Biol. Macromol..

[9] Yu Q., Nie S., Wang J., Yin P., Li W., Xie M. (2012). Polysaccharide from *Ganoderma atrum* induces tumor necrosis factor-alpha secretion via phosphoinositide 3-kinase/Akt, mitogen-activated protein kinase and nuclear factor-kappa B signaling pathways in RAW264.7 cells. Int. Immunopharmacol..

[10] Wang J., Cao B., Zhao H., Feng J. (2017). Emerging roles of *Ganoderma lucidum* in anti-aging. Aging Dis..

[11] Fu Y., Shi L., Ding K. (2019). Structure elucidation and anti-tumor activity in vivo of a polysaccharide from spores of *Ganoderma lucidum* (Fr.) Karst. Int. J. Biol. Macromol..

[12] Chen Y., Xie M., Nie S., Li C., Wang Y. (2008). Purification, composition analysis and antioxidant activity of a polysaccharide from the fruiting bodies of *Ganoderma atrum*. Food Chem..

[13] Tseng Y., Yang J., Mau J. (2008). Antioxidant properties of polysaccharides from *Ganoderma tsugae*. Food Chem..

[14] Chen X., Chen Y., Li S., Chen Y., Lan J., Liu L. (2009). Free radical scavenging of *Ganoderma lucidum* polysaccharides and its effect on antioxidant enzymes and immunity activities in cervical carcinoma rats. Carbohyd. Polym..

[15] Yang Q., Wang S., Xie Y., Sun J., Wang J. (2010). HPLC analysis of *Ganoderma lucidum* polysaccharides and its effect on antioxidant enzymes activity and Bax, Bcl-2 expression. Int. J. Biol. Macromol..

[16] Li T., Hu H., Deng W., Wu S., Wang D., Tsering T. (2015). *Ganoderma leucocontextum*, a new member of the *G. lucidum* complex from southwestern China. Mycoscience.

[17] Wang K., Bao L., Ma K., Zhang J., Chen B., Han J., Ren J., Luo H., Liu H. (2017). A novel class of alpha-glucosidase and HMG-CoA reductase inhibitors from *Ganoderma leucocontextum* and the anti-diabetic properties of ganomycin I in KK-A(y) mice. Eur. J. Med. Chem..

[18] Li X., Xie Y., Peng J., Hu H., Wu Q., Yang B.B. (2019). Ganoderiol F purified from *Ganoderma leucocontextum* retards cell cycle progression by inhibiting CDK4/CDK6. Cell Cycle.

[19] Chen H., Zhang J., Ren J., Wang W., Xiong W., Zhang Y., Bao L., Liu H. (2018). Triterpenes and meroterpenes with neuroprotective effects from *Ganoderma leucocontextum*. Chem. Biodivers..

[20] Gao X., Qi J., Ho C., Li B., Mu J., Zhang Y., Hu H., Mo W., Chen Z., Xie Y. (2020). Structural characterization and immunomodulatory activity of a water-soluble polysaccharide from *Ganoderma leucocontextum* fruiting bodies. Carbohyd. Polym..

[21] Dubois M., Gilles K.A., Hamilton J.K., Rebers P.A., Smith F. (1956). Colorimetric method for determination of sugars and related substances. Anal. Chem..

[22] Bradford M.M. (1976). Rapid and sensitive method for quantitation of microgram quantities of protein utilizing principle of protein-dye binding. Anal. Biochem..

[23] Niu Y., Shang P., Chen L., Zhang H., Gong L., Zhang X., Yu W., Xu Y., Wang Q., Yu L.L. (2014). Characterization of a novel alkali-soluble heteropolysaccharide from tetraploid *Gynostemma pentaphyllum* Makino and its potential anti-inflammatory and antioxidant properties. J. Agric. Food Chem..

[24] Chen W., Zhu X., Ma J., Zhang M., Wu H. (2019). Structural elucidation of a novel pectin-polysaccharide from the petal of *Saussurea laniceps* and the mechanism of its anti-HBV activity. Carbohyd. Polym..

[25] Re R., Pellegrini N., Proteggente A., Pannala A., Yang M., Rice-Evans C. (1999). Antioxidant activity applying an improved ABTS radical cation decolorization assay. Free Radic. Biol. Med..

[26] Xiao Y., Wang L., Rui X., Li W., Chen X., Jiang M., Dong M. (2015). Enhancement of the antioxidant capacity of soy whey by fermentation with *Lactobacillus plantarum* B1-6. J. Funct. Foods.

[27] Li W., Ji J., Chen X., Jiang M., Rui X., Dong M. (2014). Structural elucidation and antioxidant activities of exopolysaccharides from *Lactobacillus helveticus* MB2-1. Carbohyd. Polym..

[28] Benzie I., Szeto Y.T. (1999). Total antioxidant capacity of teas by the ferric reducing/antioxidant power assay. J. Agric. Food Chem..

[29] Davalos A., Gomez-Cordoves C., Bartolome B. (2004). Extending applicability of the oxygen radical absorbance capacity (ORAC-fluorescein) assay. J. Agric. Food Chem..

[30] Wang J., Ma Z., Zhang L., Fang Y., Jiang F., Phillips G.O. (2011). Structure and chain conformation of water-soluble heteropolysaccharides from *Ganoderma lucidum*. Carbohyd. Polym..

[31] Wang M., Chen G., Chen D., Ye H., Sun Y., Zeng X., Liu Z. (2019). Purified fraction of polysaccharides from Fuzhuan brick tea modulates the composition and metabolism of gut microbiota in anaerobic fermentation in vitro. Int. J. Biol. Macromol..

[32] Huo J., Wu J., Huang M., Zhao M., Sun W., Sun X., Zheng F. (2020). Structural characterization and immuno-stimulating activities of a novel polysaccharide from *Huangshui*, a byproduct of Chinese Baijiu. Food Res. Int..

[33] Zhou W., Zhao Y., Yan Y., Mi J., Lu L., Luo Q., Li X., Zeng X., Cao Y. (2020). Antioxidant and immunomodulatory activities in vitro of polysaccharides from bee collected pollen of Chinese wolfberry. Int. J. Biol. Macromol..

[34] Liu Z., Jiao Y., Lu H., Shu X., Chen Q. (2020). Chemical characterization, antioxidant properties and anticancer activity of exopolysaccharides from *Floccularia luteovirens*. Carbohyd. Polym..

[35] Wang Y., Yin J., Huang X., Nie S. (2020). Structural characteristics and rheological properties of high viscous glucan from fruit body of *Dictyophora rubrovolvata*. Food Hydrocoll..

[36] Zhang J., Chen M., Wen C., Zhou J., Gu J., Duan Y., Zhang H., Ren X., Ma H. (2019). Structural characterization and immunostimulatory activity of a novel polysaccharide isolated with subcritical water from *Sagittaria sagittifolia* L.. Int. J. Biol. Macromol..

[37] Cheng Y., Xiao X., Li X., Song D., Lu Z., Wang F., Wang Y. (2017). Characterization, antioxidant property and cytoprotection of exopolysaccharide-capped elemental selenium particles synthesized by *Bacillus paralicheniformis* SR14. Carbohyd. Polym..

[38] Kpodo F.M., Agbenorhevi J.K., Alba K., Bingham R.J., Oduro I.N., Morris G.A., Kontogiorgos V. (2017). Pectin isolation and characterization from six okra genotypes. Food Hydrocoll..

[39] Liu C., Cheung P.C.K. (2019). Structure and immunomodulatory activity of microparticulate mushroom sclerotial beta-glucan prepared from *Polyporus rhinoceros*. J. Agric. Food Chem..

[40] Gu J., Zhang H., Wen C., Zhang J., He Y., Ma H., Duan Y. (2020). Purification, characterization, antioxidant and immunological activity of polysaccharide from *Sagittaria sagittifolia* L.. Food Res. Int..

[41] Rozi P., Abuduwaili A., Mutailifu P., Gao Y., Rakhmanberdieva R., Aisa H.A., Yili A. (2019). Sequential extraction, characterization and antioxidant activity of polysaccharides from *Fritillaria pallidiflora* Schrenk. Int. J. Biol. Macromol..

[42] Qian J., Chen W., Zhang W., Zhang H. (2009). Adulteration identification of some fungal polysaccharides with SEM, XRD, IR and optical rotation: A primary approach. Carbohyd. Polym..

[43] Kong L., Yu L., Feng T., Yin X., Liu T., Dong L. (2015). Physicochemical characterization of the polysaccharide from *Bletilla striata*: Effect of drying method. Carbohyd. Polym..

[44] Marszalek P.E., Dufrene Y.F. (2012). Stretching single polysaccharides and proteins using atomic force microscopy. Chem. Soc. Rev..

[45] Deng Y., Li M., Chen L., Chen X., Lu J., Zhao J., Li S. (2018). Chemical characterization and immunomodulatory activity of acetylated polysaccharides from *Dendrobium devonianum*. Carbohyd. Polym..

[46] Wang K., Wang J., Li Q., Zhang Q., You R., Cheng Y., Luo L., Zhang Y. (2014). Structural differences and conformational characterization of five bioactive polysaccharides from *Lentinus edodes*. Food Res. Int..

[47] Giannotti M.I., Rinaudo M., Vancso G.J. (2007). Force spectroscopy of hyaluronan by atomic force microscopy: From hydrogen-bonded networks toward single-chain behavior. Biomacromolecules.

[48] Li J., Yuan P., Wang X., Aipire A., Li M., Yang J., Tao H., Ying T., Fu C., Wei X. (2017). Purification, characterization and bioactivities of polysaccharides from *Pleurotus ferulae*. Food Funct..

[49] Lopez-Alarcon C., Denicola A. (2013). Evaluating the antioxidant capacity of natural products: A review on chemical and cellular-based assays. Anal. Chim. Acta.

[50] Shahidi F., Ambigaipalan P. (2015). Phenolics and polyphenolics in foods, beverages and spices: Antioxidant activity and health effects - A review. J. Funct. Foods.

[51] Liu W., Wang H., Pang X., Yao W., Gao X. (2010). Characterization and antioxidant activity of two low-molecular-weight polysaccharides purified from the fruiting bodies of *Ganoderma lucidum*. Int. J. Biol. Macromol..

[52] Shang H., Zhou H., Duan M., Li R., Wu H., Lou Y. (2018). Extraction condition optimization and effects of drying methods on physicochemical properties and antioxidant activities of polysaccharides from comfrey (*Symphytum officinale* L.) root. Int. J. Biol. Macromol..

[53] Cai L., Zou S., Liang D., Luan L. (2018). Structural characterization, antioxidant and hepatoprotective activities of polysaccharides from *Sophorae tonkinensis* Radix. Carbohyd. Polym..

[54] Huang S., Ding S., Fan L. (2012). Antioxidant activities of five polysaccharides from *Inonotus obliquus*. Int. J. Biol. Macromol..

[55] Song H., Zhang Q., Zhang Z., Wang J. (2010). In vitro antioxidant activity of polysaccharides extracted from *Bryopsis plumose*. Carbohyd. Polym..

[56] Jiang L., Wang W., Wen P., Shen M., Li H., Ren Y., Xiao Y., Song Q., Chen Y., Yu Q. (2020). Two water-soluble polysaccharides from mung bean skin: Physicochemical characterization, antioxidant and antibacterial activities. Food Hydrocoll..

[57] Wang Z., Yi K., Lin Q., Yang L., Chen X., Chen H., Liu Y., Wei D. (2019). Free radical sensors based on inner-cutting graphene field-effect transistors. Nat. Commun..

[58] Poljsak B., Suput D., Milisav I. (2013). Achieving the balance between ROS and antioxidants: When to use the synthetic antioxidants. Oxid. Med. Cell. Longev..

[59] Pereira-Caro G., Sarria B., Madrona A., Espartero J.L., Goya L., Bravo L., Mateos R. (2011). Alkyl hydroxytyrosyl ethers show protective effects against oxidative stress in HepG2 cells. J. Agric. Food Chem..

[60] Zhang X., Wang L., Wang R., Luo X., Li Y., Chen Z. (2016). Protective effects of rice dreg protein hydrolysates against hydrogen peroxide-induced oxidative stress in HepG-2 cells. Food Funct..

[61] Lue J., Lin P.H., Yao Q., Chen C. (2010). Chemical and molecular mechanisms of antioxidants: Experimental approaches and model systems. J. Cell. Mol. Med..

[62] Brigelius-Flohe R., Maiorino M. (2013). Glutathione peroxidases. Biochim. Biophys. Acta Gen. Subj..

[63] Li Q., Feng Y., He W., Wang L., Wang R., Dong L., Wang C. (2017). Post-screening characterisation and in vivo evaluation of an anti-inflammatory polysaccharide fraction from *Eucommia ulmoides*. Carbohyd. Polym..

[64] Zhan Q., Wang Q., Lin R., He P., Lai F., Zhang M., Wu H. (2020). Structural characterization and immunomodulatory activity of a novel acid polysaccharide isolated from the pulp of *Rosa laevigata* Michx fruit. Int. J. Biol. Macromol..

[65] Ru Y., Chen X., Wang J., Guo L., Lin Z., Peng X., Qiu B. (2019). Polysaccharides from *Tetrastigma hemsleyanum Diels et Gilg*: Extraction optimization, structural characterizations, antioxidant and antihyperlipidemic activities in hyperlipidemic mice. Int. J. Biol. Macromol..

[66] Li J., Gu F., Cai C., Hu M., Fan L., Hao J., Yu G. (2020). Purification, structural characterization, and immunomodulatory activity of the polysaccharides from *Ganoderma lucidum*. Int. J. Biol. Macromol..

[67] Zhang M., Wang G., Lai F., Wu H. (2016). Structural characterization and immunomodulatory activity of a novel polysaccharide from *Lepidium meyenii*. J. Agric. Food Chem..

