# Supplementary figures and images for: Purification, Physicochemical Properties, and Antioxidant Activities of Two Low-Molecular-Weight Polysaccharides from Ganoderma leucocontextum Fruiting Bodies
Source: Antioxidants (Basel). 2021 Jul 20;10(7):1145. doi: 10.3390/antiox10071145 (PMC8301108; doi:10.3390/antiox10071145)

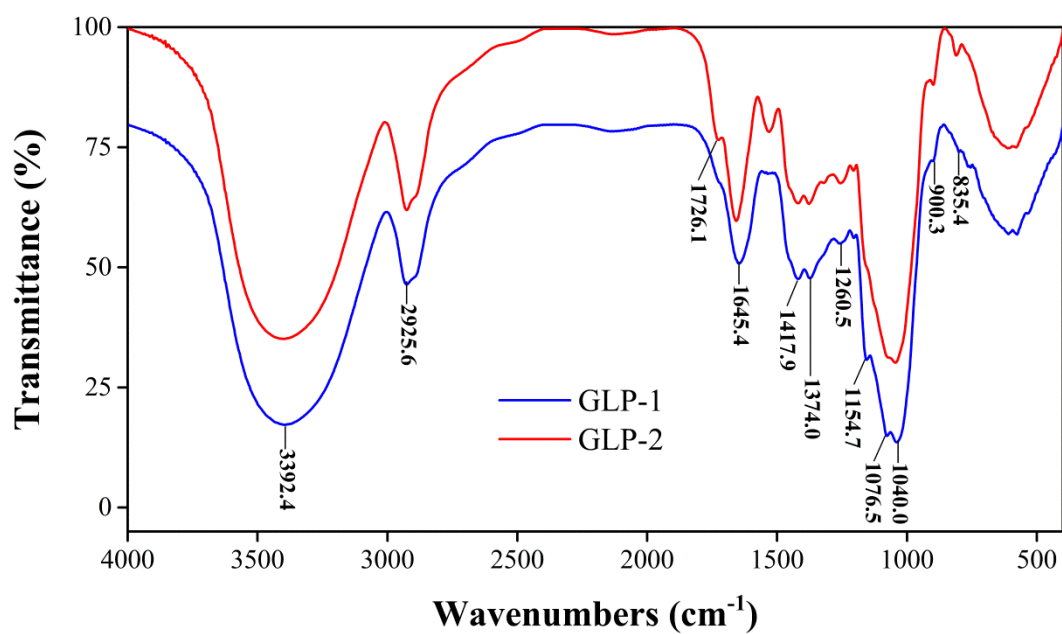

**Figure S1.** FT-IR spectra of GLP-1 and GLP-2.

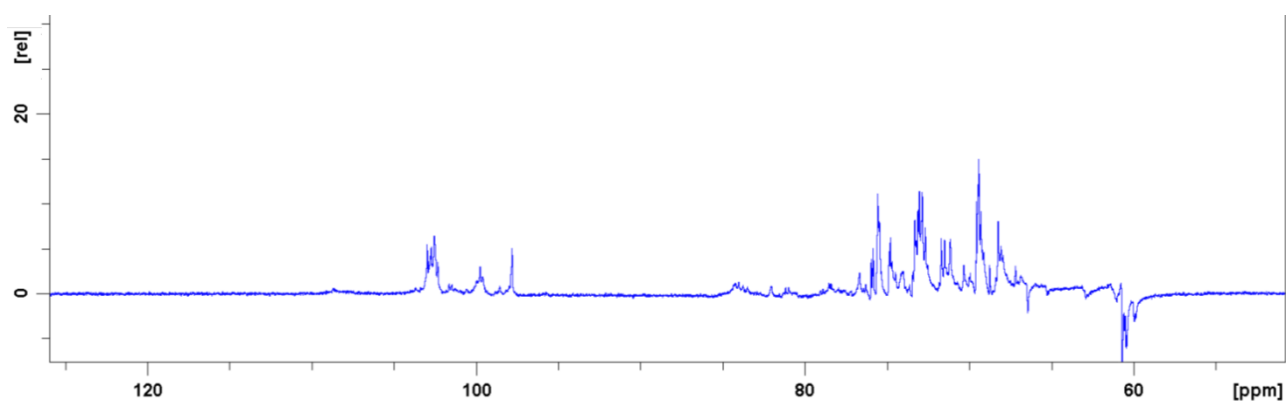

**Figure S2.** DEPT135 spectrum of GLP-1.

Supplement: Supplementary file 1 [file antioxidants-10-01145-s001.zip › antioxidants-1292950-supplementary.pdf]
